# Effect of Host Moieties on the Phosphorescent Spectrum of Green Platinum Complex

**DOI:** 10.3390/molecules24030454

**Published:** 2019-01-28

**Authors:** Yukiko Iwasaki, Hirohiko Fukagawa, Takahisa Shimizu

**Affiliations:** Japan Broadcasting Corporation (NHK), Science & Technology Research Laboratories, 1-10-11 Kinuta, Setagaya-ku, Tokyo 157-8510, Japan; fukagawa.h-fe@nhk.or.jp (H.F.); shimizu.t-li@nhk.or.jp (T.S.)

**Keywords:** OLED, phosphorescent, high color purity, platinum complex

## Abstract

Highly efficient, operationally stable, and pure-color organic light-emitting diodes (OLEDs) are of considerable significance for developing practical wide-color-gamut displays. Further, we have demonstrated the feasibility of an efficient pure green phosphorescent OLED (PHOLED) by employing a narrow-band platinum complex and a top-emitting structure. The utilization of the thermally activated delayed fluorescence (TADF) material as the phosphorescent host is expected to serve as a promising solution for obtaining operationally stable PHOLEDs with high color purity. However, the emission spectrum of the platinum complex in the TADF host exhibits a considerably broad emission spectrum. This study investigates the cause of the spectral change by evaluating the photoluminescence spectra of the platinum complex in various hosts exhibiting different molecular structures. The triazine unit in the host material was observed to result in exciplex formation between the lowest unoccupied molecular orbital (LUMO) of the host and the highest occupied molecular orbital (HOMO) of the platinum complex. Therefore, the TADF material that sterically hinders the triazine unit is considered to be suitable to prevent both exciplex formation and spectral broadening.

## 1. Introduction

Light-emitting devices with a narrow bandwidth are required for developing wide-color-gamut (WCG) displays. Recently, various types of devices, such as organic light-emitting diodes (OLEDs) [1,2,3], quantum dot LEDs (QD-LEDs) [4,5], and perovskite LEDs [6,7] with high color purity, have been reported. The wide-color gamut with RGB primaries positioned on the spectral locus is specified in Recommendation ITU-R BT.2020 (BT.2020) for ultra-high-definition television (UHDTV) [8]. The color gamut of UHDTV is wider than that of high-definition television which is specified in Recommendation ITU-R BT.709 [9]; therefore, it is essential for the display devices to exhibit improved color purity to realize UHDTV displays that are compliant with BT.2020.

Recently, OLEDs have become commercially available in products, including large-sized TVs and mobile phones. Although highly efficient OLEDs can be obtained using phosphorescent [10,11] or thermally activated delayed fluorescence (TADF) emitters [12], it is considered difficult to achieve emission with high color purity in OLEDs because of the broad emission spectrum of organic compounds. However, by employing a planar and rigid molecular structure, high color purity has been obtained in the platinum complex [1,2] and the TADF emitter [13]. Further, we reported the feasibility of an efficient and pure green phosphorescent OLED (PHOLED) with the CIE 1931 color coordinates of (0.18, 0.74) and a current efficiency of 98 cd A^−1^ by employing both the narrow-band phosphorescent emitter platinum(II)-2′-(3-methylimidazol-1-yl)-9-(pyridine-2-yl)-9*H*-2,9′-bicarbazole (PtN7N) [2] and a top-emitting structure [14]. Even though some emission peaks could be observed in the emission of PtN7N, the effect of vibrational structures was suppressed by the fabrication of the top-emitting device having a microcavity structure. Further, we demonstrated an efficient pure green OLED, whose color coordinates were close to BT.2020 green. Along with efficiency and color purity, a high operational stability is considered to be essential for practical WCG OLED displays. Our previous study has indicated that the device performance is strongly dependent on the host material in the emitting layer (EML) [14]. Although the pure green PHOLED using 2,7-diphenyl-4*b*-*aza*-12*b*-boradibenzo[*g*,*p*]chrysene (BN–DBC–Ph_2_) as a host exhibited higher efficiency and operational stability than that exhibited by the PHOLED using the conventional host 4,4′-bis(9-carbazolyl)-2,2′-biphenyl (CBP), it exhibited an operational lifetime, i.e., the time for the luminance to decay to 50% of the initial luminance (LT50) [15], of less than 2000 h with an initial luminance of 1000 cd m^−2^, which is considered to be insufficient for practical use. Therefore, the lifetime of the pure green PHOLED is considered to be the final issue for the practical application.

Further, the operational stability of PHOLEDs can be improved by selecting suitable host materials; the configurations of the EMLs that can be used to obtain operationally stable PHOLEDs have been proposed over the recent years [16,17,18,19,20]. Highly efficient and operationally stable PHOLEDs have been demonstrated using the following two suitable hosts: one is an exciplex with a small energy gap between the singlet (S_1_) and triplet (T_1_) excited states (∆E_ST_) [16,19], whereas the other is a single TADF material comprising both donor and acceptor units [17,18]. The configuration in which an EML employs a phosphorescent emitter and in which the TADF material serves as a host is considered to be suitable for obtaining low-cost and high-performance PHOLEDs because it considerably reduces the concentration of expensive phosphorescent dopants to 1–3 wt%. The low concentration of the phosphorescent emitter is considered to be particularly advantageous for the expensive platinum complex emitter. Actually, we demonstrated that the lifetime of PHOLEDs employing the TADF material 2,4-diphenyl-6-bis(12-phenylindolo)[2,3-*a*]carbazole-11-yl)-1,3,5-triazine (DIC–TRZ) as a host of *fac*-tris(3-methyl-2-phenylpyridinato-*N,C2*′-)iridium(III) (Ir(mppy)_3_) exhibited a LT50 of 14,000 h, which is approximately 27 times longer than that of PHOLEDs using CBP [20]. DIC–TRZ is a donor–acceptor-type TADF material comprising indolocarbazole and triazine units [21]. For realizing pure-color, highly efficient, and operationally stable PHOLEDs, an emitting layer that comprises TADF materials and PtN7N is considered to be effective. However, the usage of the TADF material as the phosphorescent host causes color-purity issues. The emission spectrum of the 1 wt% PtN7N-doped deposited film in DIC–TRZ is broader than that in CBP. Further, the broadened EML emission peaks can decrease the external quantum efficiency (EQE) of the OLED displays by employing a top-emitting structure and/or a color filter. Furthermore, it is essential to clarify the effect of host materials on the emission spectrum of phosphorescent emitters with narrow emission bands, such as PtN7N, to develop pure green PHOLEDs with high efficiency and high operational stability.

This study investigates the cause of the spectral change of PtN7N by evaluating the photoluminescence (PL) spectra of the EMLs comprising several PtN7N-host combinations. First, the effect of the acceptor moieties in host materials on the emission spectrum is investigated, which indicates that the triazine unit causes a spectral change. Subsequently, the emission spectra of the EMLs are investigated using host materials with different energy levels, indicating that the spectral change is caused by the exciplex formation between the host and PtN7N. Furthermore, this interaction was successfully prevented by employing host materials with a steric hindrance surrounding the triazine unit. These observations indicate that the TADF materials with a steric hindrance around the triazine unit are considered to be suitable host materials for obtaining pure-color, highly efficient, and operationally stable PHOLEDs.

## 2. Results and Discussion

### 2.1. Effect of the Acceptor Moieties in Host Materials on the Emission Spectrum of PtN7N

**Figure 1:**
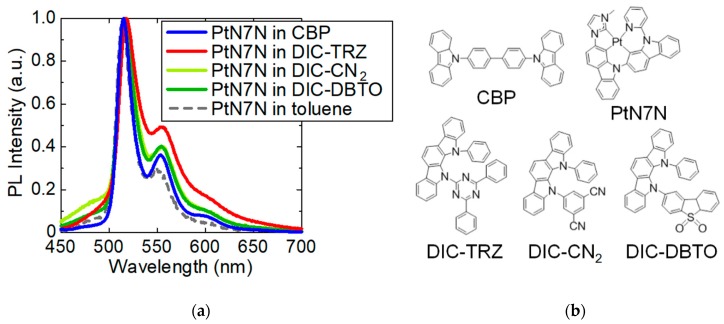
PL spectra and molecular structures of PtN7N in different host materials such as CBP, DIC–TRZ, and toluene (1 × 10^−5^ M): (**a**) the normalized PL spectra and (**b**) the molecular structures.

**Figure 2:**
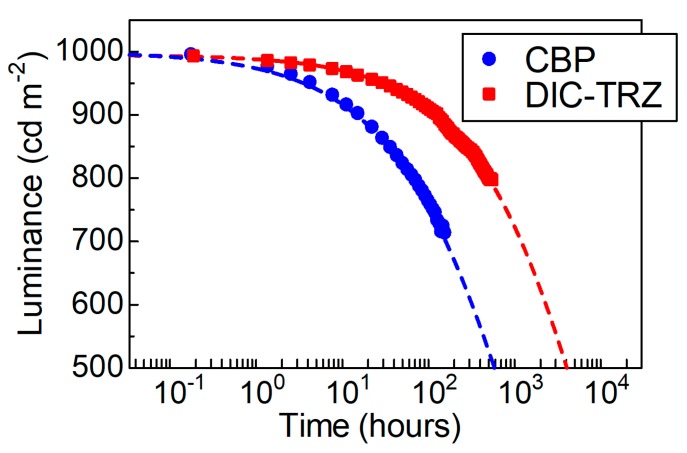
Luminance–time characteristics for the PtN7N devices fabricated using different host materials under a constant current with an initial luminance of 1000 cd m^−2^.

First, the origin of spectral broadening is investigated using DIC–TRZ as the host material. Figure 1 shows the PL spectra of the 1-wt% PtN7N-doped deposited films in both DIC–TRZ and CBP [14], and their molecular structures. The emission spectrum of a PtN7N-doped-DIC–TRZ film is observed to be broader than the solution in toluene. Furthermore, a PHOLED with an EML comprising DIC–TRZ and PtN7N is fabricated (see Supplementary Materials Figure S1). Figure 2 shows the luminance–time characteristics that have been measured under constant dc with an initial luminance of 1000 cd m^−2^. Even though the EQE of the PHOLED using DIC–TRZ was lower than that observed using CBP (see Supplementary Materials Figure S2), the expected LT50 of the PHOLED using DIC–TRZ was 4000 h, which was 6.7 times longer than that obtained using CBP. Although the emission spectrum of the PHOLED that was obtained using DIC–TRZ as the host material was broad, the TADF materials are confirmed to be effective for achieving operationally stable PHOLEDs that employ PtN7N. Further, the acceptor structures are considered to be one of the origins of the spectral change because spectral broadening could not be observed using CBP, which mainly comprised donor carbazoles. Thus, the PL spectra of the 1 wt% PtN7N-doped films are evaluated using several hosts containing indolocarbazole and various acceptors. Further, the normalized PL spectra of the films and molecular structures of the host materials are shown in Figure 1. 

6-Bis(12-phenylindolo)[2,3-a]carbazole-11-yl)-3,5-dicarbonitrile (DIC–CN_2_), 6-bis(12-phenyl-indolo)[2,3-a]-carbazole-11-yl)-2-dibenzothiophene sulfone (DIC–DBTO), and DIC–TRZ were used as the host materials. As shown in Figure 1a, the spectral shapes of PtN7N in DIC–CN_2_ and DIC–DBTO were approximately the same and their peak intensity of PtN7N was low at ~560 nm (ca. 0.40). Conversely, in DIC–TRZ, the peak intensity at ~560 nm was higher (ca. 0.49) than the solution in toluene. Although the highest occupied molecular orbital (HOMO) level and the lowest unoccupied molecular orbital (LUMO) levels of DIC–TRZ are the intermediate values between the values of DIC–CN_2_ and DIC–DBTO, respectively (see Supplementary Materials Figure S3), only the PtN7N in DIC–TRZ exhibited a relatively broad emission spectrum. Based on these results, spectral broadening is considered to have been caused by the triazine acceptor moiety in DIC–TRZ.

### 2.2. Verification of the Spectral Change by Employing Host Materials Without Carbazole Units

**Figure 3:**
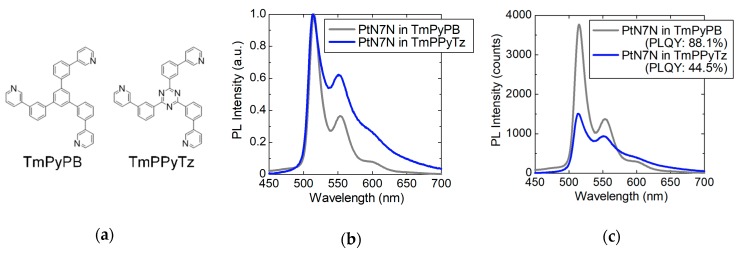
The molecular structures and PL spectra of PtN7N in TmPyPB and TmPPyTz: (**a**) molecular structures; (**b**) normalized PL spectra; and (**c**) non-normalized PL spectra.

In this study, we investigated the PL spectra of the 1-wt% PtN7N-doped films in host materials without carbazole units to verify the effect of the triazine unit on the emisson spectrum of PtN7N. Figure 3a shows the molecular structures of the host materials (1,3,5-tri[(3-pyridyl)-phenyl-3-yl] benzene (TmPyPB) and 2,4,6-tris(*m*-pyridin-3-yl-phenyl)triazine (TmPPyTz)). Their molecular structures are exactly identical except for the center units. Further, the PL spectra of PtN7N in TmPyPB and TmPPyTz are shown in Figure 3b,c, respectively. Figure 3b shows the PL spectra normalized to the main peak, whereas Figure 3c shows the non-normalized PL spectra. Further, the PL spectrum of PtN7N in TmPPyTz exhibits extremely broad emission. In Figure 3c, we can observe a decrease in the intensity of the peaks at ca. 515 and 560 nm as well as an increase in the intensity of a new peak at a long wavelength, i.e., at more than 600 nm, when the triazine is used as the center unit. In addition, the photoluminescence quantum yield (PLQY) of the PtN7N-doped TmPyPB film was observed to be 88.1%, whereas that of the TmPPyTz film was observed to be 44.5%. Thus, we indicate that the spectral change originates from the exciplex formation between the host and PtN7N and that it was only formed when the host material contained a triazine unit.

### 2.3. Verification of the Spectral Change by Employing Host Materials With Different Energy Levels

**Figure 4:**
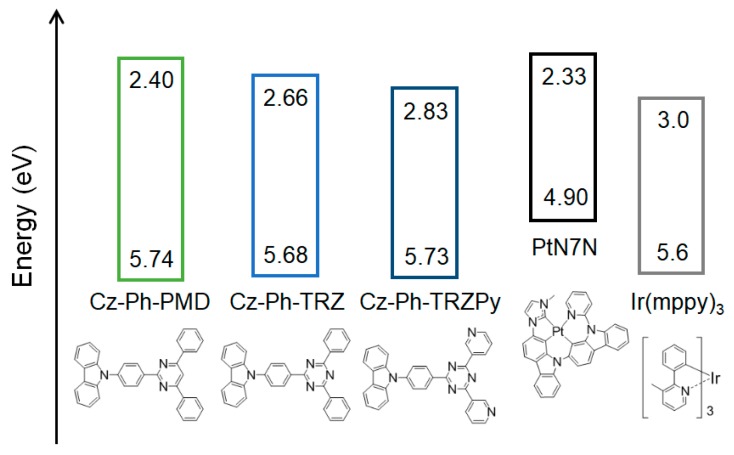
Molecular structures and energy levels of the host materials and emitters.

**Figure 5:**
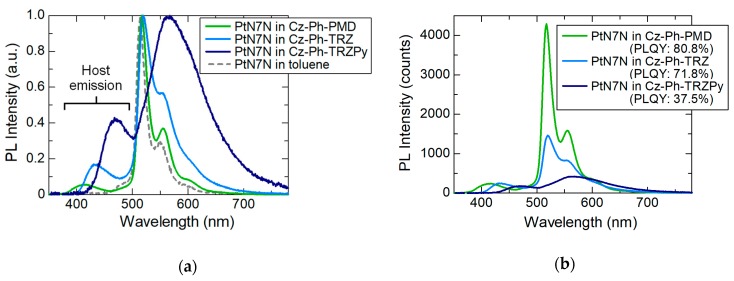
PL spectra of PtN7N in Cz-Ph-PMD, Cz-Ph-TRZ and Cz-Ph-TRZPy: (**a**) the normalized PL spectra and (**b**) the non-normalized PL spectra.

To confirm the exciplex formation between the host and PtN7N, similar host materials with different energy levels were used. The molecular structures of these host materials are shown in Figure 4. To systematically understand the relation between the energy levels and spectral changes, we compared three host materials, including (9-(4-(4,6-diphenylpyrimidin-2-yl)phenyl)-9*H*- carbazole (Cz–Ph–PMD), 9-(4-(4,6-diphenyl-1,3,5-triazin-2-yl)phenyl)-9*H*-carbazole (Cz–Ph–TRZ), and 9-(4-(4,6-tris(2-pyridyl)-1,3,5-triazin-2-yl)phenyl)-9*H*-carbazole (Cz–Ph–TRZPy)), with donor–acceptor structures comprising carbazoles and acceptors having different number of nitrogen atoms. Further, the measured energy levels of these host materials and PtN7N are shown in Figure 4. The HOMO levels are observed to be approximately similar in all the host materials. However, the LUMO level becomes lower with increasing number of nitrogen atoms in the acceptor. Additionally, the HOMO level of PtN7N is considerably higher than that of conventional emitters such as Ir(mppy)_3_. The normalized PL spectra of the 1 wt% PtN7N-doped films in these host materials are shown in Figure 5a. As exhibited in Figure 5a, the usage of Cz–Ph–PMD with a pyrimidine as an acceptor moiety resulted in narrow-band emission. However, broader emissions were observed when host materials with a triazine were used as an acceptor moiety. Further, lower LUMO levels of the host materials led to broad emission. The non-normalized host-dependent PL spectra are shown in Figure 5b. As can be observed in Figure 3c, there was an obvious decrease in the intensity of the peaks at approximately 515 and 560 nm; also, a new peak can be observed at a wavelength that is slightly more than 600 nm. The PLQY also decreased when the intensity of the main peak of PtN7N decreased at approximately 515 nm (Figure 5b). These results indicate that exciplexes are formed between the HOMO level of PtN7N and the LUMO level of the host material. Further, exciplexes are considered to be actively formed when the energy difference between the HOMO level of PtN7N and the LUMO level of the host material is small. 

**Figure 6:**
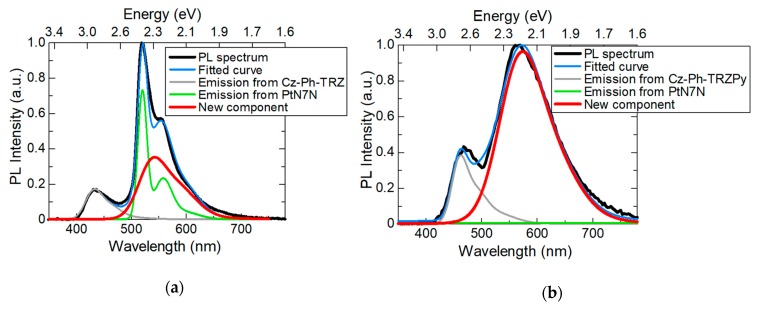
Deconvolution of the peaks in the PL spectra for the (**a**) PtN7N-doped Cz–Ph–TRZ film and (**b**) PtN7N-doped Cz–Ph–TRZPy film.

To examine the exciplexes that are formed between the LUMO of the host and the HOMO of PtN7N, the PL spectra of the PtN7N-doped Cz–Ph–TRZ and Cz–Ph–TRZPy films are deconvoluted, as shown in Figure 6a,b, respectively. The PL spectrum of the Cz–Ph–TRZ film is considered to be a combination of the following three components: the emission from the host, PtN7N, and the new component. The PL spectrum of the Cz–Ph–TRZPy film comprises the following two components: the emission from the host and the new component. The energy of the emission peak of the new components was approximately 2.29 and 2.16 eV in the PtN7N-doped Cz–Ph–TRZ and the PtN7N-doped Cz–Ph–TRZPy films, respectively. Further, the difference between the energies of the new component peaks (0.13 eV) is almost equal to the energy difference between the LUMO levels of the hosts (0.17 eV, see Figure 4). Therefore, the emission spectrum that was derived from the exciplex formation exhibited a red shift when the LUMO levels became lower.

### 2.4. Effect of Steric Hindrance in the Host Materials on the Emission Spectrum of PtN7N

**Figure 7:**
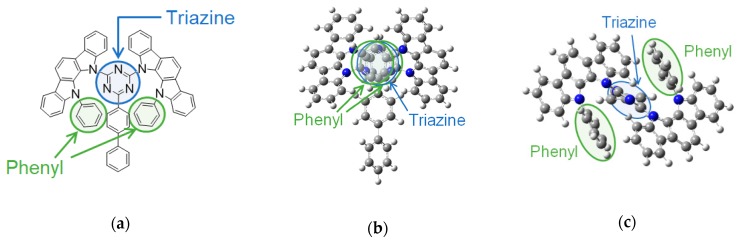
PIC–TRZ: (**a**) molecular structure; (**b**) optimized molecular structure (from the side); and (**c**) optimized molecular structure (from the top).

**Figure 8:**
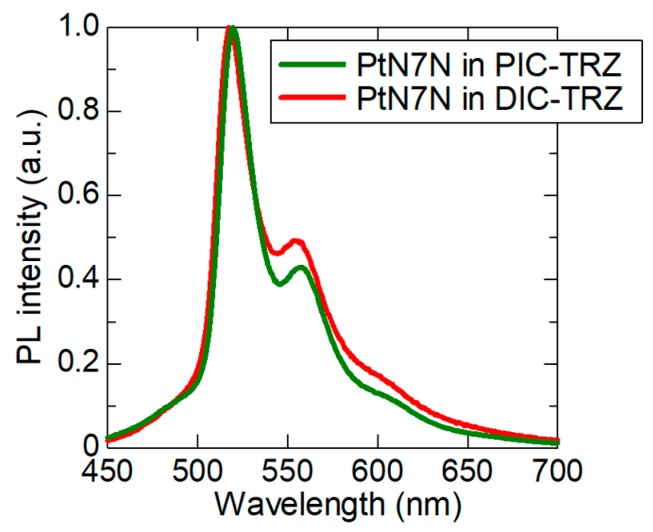
Normalized PL spectra of PtN7N in PIC–TRZ and DIC–TRZ.

The interaction between two moieties of the related molecules causes exciplex formation [22]. In Section 2.1 and Section 2.2, we conjectured that the triazine unit in the host material is considered to be the origin of the spectral change. In Section 2.3, the LUMO levels of the host materials also affect the normalized PL spectra, particularly the peak intensity at ~560 nm. We suggest that an encounter complex is formed between the molecular orbitals, which are distributed on the triazine unit of the host, and PtN7N; further, the spectral change is caused by this exciplex formation. To verify our hypothesis, we measured the PL spectrum of the PtN7N-doped 2-biphenyl-4,6-bis(12-phenylindolo [2,3-*a*]carbazol-11-yl)-1,3,5-triazine (PIC–TRZ) [23] film. In the optimized molecular structures, as shown in Figure 7b,c, two phenyl units surrounding the triazine unit in PIC–TRZ, resulting in steric hindrance. The normalized PL spectrum of the 1 wt% PtN7N-doped PIC–TRZ film is shown in Figure 8. Further, the normalized PL peak intensity at ~560 nm is ~0.43, which is relatively lower than obtained using DIC–TRZ.

This indicates that the steric hindrance surrounding the triazine unit in the host material has suppressed the exciplex formation and has prevented the spectral change. An increase in the intensity at ~560 nm derived from the exciplex emission and a decrease in the peak intensity at ~515 nm derived from PtN7N emission were suppressed by employing PIC–TRZ as a host material. Therefore, the normalized PL spectrum of PIC–TRZ film shows a lower PL intensity at ~560 nm than that of DIC–TRZ film.

To develop highly efficient and pure green PHOLEDs with high operational stability, the TADF materials with donors and acceptors are considered to be suitable hosts. However, they should be designed to avoid undesirable exciplex formation between the triazine unit of the host material and PtN7N. Therefore, this study proposes a design of TADF materials such that the triazine unit is subjected to steric hindrance; this is considered be a suitable strategy for preventing the spectral change in the emitter to obtain pure-color, highly efficient, and operationally stable PHOLEDs.

## 3. Materials and Methods

### 3.1. Fabrication of OLEDs

The OLEDs were fabricated on the glass substrates coated with a 100 nm thick indium tin oxide layer as the anode. Before fabricating the organic layers, the substrate was cleaned using ultra-purified water and organic solvents and was treated with UV-ozone in ambient conditions. To reduce the possibility of electrical shorts within the device, a 30 nm thick layer of Clevios HIL 1.3 N (supplied by Heraeus Holding GmbH, Hanau, Germany) was spin coated onto the substrate.

Further, the organic layers were sequentially deposited onto the substrate without breaking the vacuum at ~10^−5^ Pa. The film structures of the PHOLEDs using CBP and DIC–TRZ were obtained as follows: α-NPD (20 nm)/4DBFP3Q [24] (10 nm)/host:PtN7N (6 wt%, 25nm)/TPBi (35 nm), where α-NPD represents 4,4′-bis[*N*-(1-naphthyl)-*N*-phenyl-amino]biphenyl, 4DBFP3Q represents *N*3,*N*3‴-bis(dibenzo[*b*,*d*] furan-4-yl)-*N*3,*N*3‴-diphenyl-[1,1′:2′,1″:2″,1‴-quaterphenyl]-3,3‴-diamine and TPBi represents 1,3,5-tris(*N*-phenylbenzimidazol-2-yl)benzene. After the formation of the organic layers, a 0.8 nm thick LiF layer and a 100 nm thick Al layer were deposited that served as cathode. The devices were further encapsulated using a UV-epoxy resin and a glass cover in a nitrogen atmosphere.

### 3.2. Photoluminescence Measurement

The 50 nm thick organic films were fabricated on clean quartz glass substrates using thermal evaporation. Further, the PL spectra and PLQY of the organic films were measured using a PLQY measurement system (Quantaurus-QY, Hamamatsu Photonics, Shizuoka, Japan) at 300 K. The excitation wavelength was observed to be 300 nm in all the PL measurements was 300 nm.

### 3.3. Energy Level Measurement

The HOMO levels were measured using photoelectron spectroscopy (AC-3, Rikenkeiki, Tokyo, Japan). The LUMO levels were estimated by subtracting the optical band gap energy from the HOMO levels.

### 3.4. Calculation of the Optimized Structures

The optimized structures were calculated using the Gaussian09 program with B3LYP/6-31G (d,p) basis sets.

## 4. Conclusions

We investigated the cause of spectral change in the phosphorescent spectrum of the green platinum complex using various hosts. Even though the TADF materials with donors and acceptors are considered to be suitable hosts for operationally stable PHOLEDs, the emission spectrum of the platinum complex in a TADF host exhibits a considerably broad emission spectrum. We found that the triazine unit in the host material caused the spectral change. Further, we verified the emission spectrum of PtN7N using host materials with different energy levels and revealed that the exciplex formation between the LUMO of the host and the HOMO of PtN7N was the cause of spectral broadening. To ensure that the spectrum of PtN7N is narrow, designing TADF materials with a steric hindrance surrounding the triazine acceptor moiety is considered to be an effective strategy. These observations will contribute to the realization of pure-color, highly efficient, and operationally stable PHOLEDs.

## Supplementary Materials

The following is available online, Figure S1: Energy level diagram of the phosphorescent organic light-emitting diodes; Figure S2: Performance of the phosphorescent organic light-emitting diodes with different host materials such as CBP and DIC–TRZ: (a) the luminance–voltage characteristics and (b) the external quantum efficiency–current density curves; Figure S3: The HOMO and LUMO levels of the host materials (CBP, DIC–TRZ, DIC–CN_2_, DIC–DBTO, and PIC–TRZ) and the emitter (PtN7N); Table S1: Full width at half maximum (FWHM) values of the photoluminescence spectra of the 0–0 band of PtN7N in different host materials such as CBP, DIC–TRZ, DIC–CN_2_, DIC–DBTO, TmPyPB, TmPPyTz, Cz–Ph–PMD, Cz–Ph–TRZ, Cz–Ph–TRZPy, and PIC–TRZ.
