# Supplementary material for: Effect of Host Moieties on the Phosphorescent Spectrum of Green Platinum Complex
Source: Molecules. 2019 Jan 28;24(3):454. doi: 10.3390/molecules24030454 (PMC6384832; doi:10.3390/molecules24030454)
Supplement: Supplementary file 1 [file molecules-24-00454-s001.pdf]

Supplementary Materials

Effect of Host Moieties on the Phosphorescent Spectrum of Green Platinum Complex

Yukiko Iwasaki, Hirohiko Fukagawa and Takahisa Shimizu

Japan Broadcasting Corporation (NHK), Science & Technology Research Laboratories, 1-10-11 Kinuta, Setagaya-ku, Tokyo 157-8510, Japan

Figure Captions:

Figure S1: Energy level diagram of the phosphorescent organic light-emitting diodes.

Figure S2: Performance of the phosphorescent organic light-emitting diodes with different host materials such as CBP and DIC–TRZ: (a) the luminance–voltage characteristics and (b) the external quantum efficiency–current density curves.

Figure S3: The HOMO and LUMO levels of the host materials (CBP, DIC–TRZ, DIC–CN_2, DIC–DBTO, and PIC–TRZ) and the emitter (PtN7N).

Table S1: Full width at half maximum (FWHM) values of the photoluminescence spectra of the 0–0 band of PtN7N in different host materials such as CBP, DIC–TRZ, DIC–CN_2, DIC–DBTO, TmPyPB, TmPPyTz, Cz–Ph–PMD, Cz–Ph–TRZ, Cz–Ph–TRZPy, and PIC–TRZ.

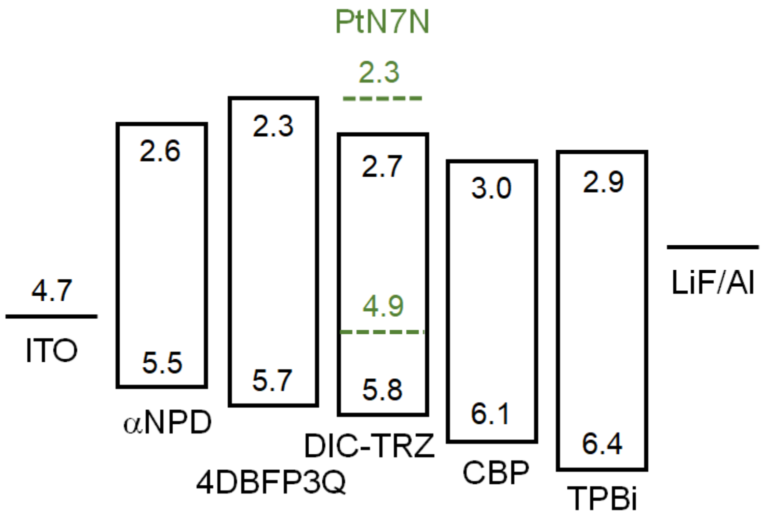

Figure S1. Energy level diagram of the phosphorescent organic light-emitting diodes.

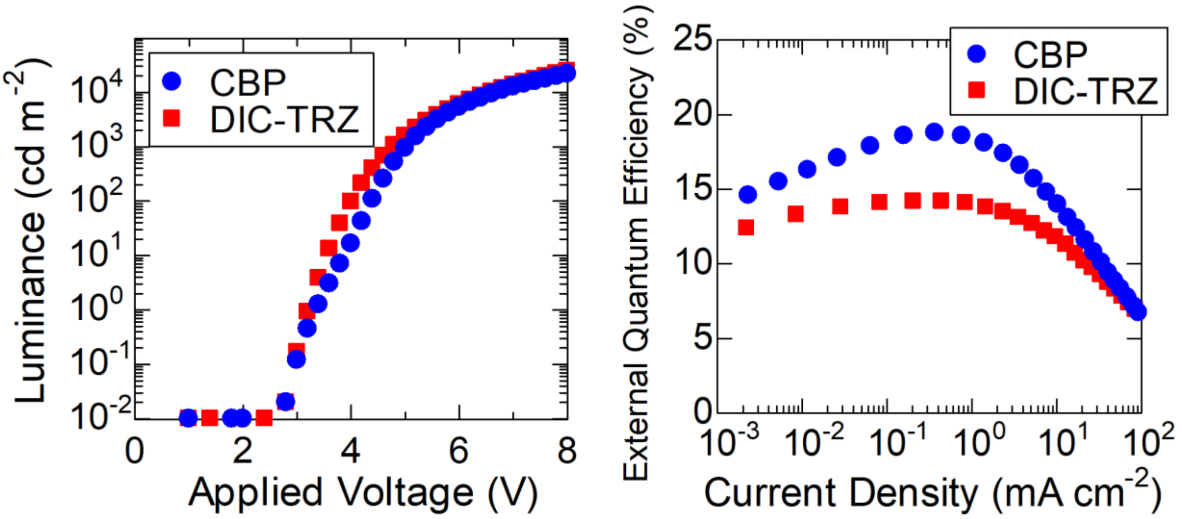

(**a**) (**b**)

Figure S2. Performance of the phosphorescent organic light-emitting diodes with different host materials, such as CBP and DIC–TRZ: (a) the luminance–voltage characteristics and (b) the external quantum efficiency–current density curves.

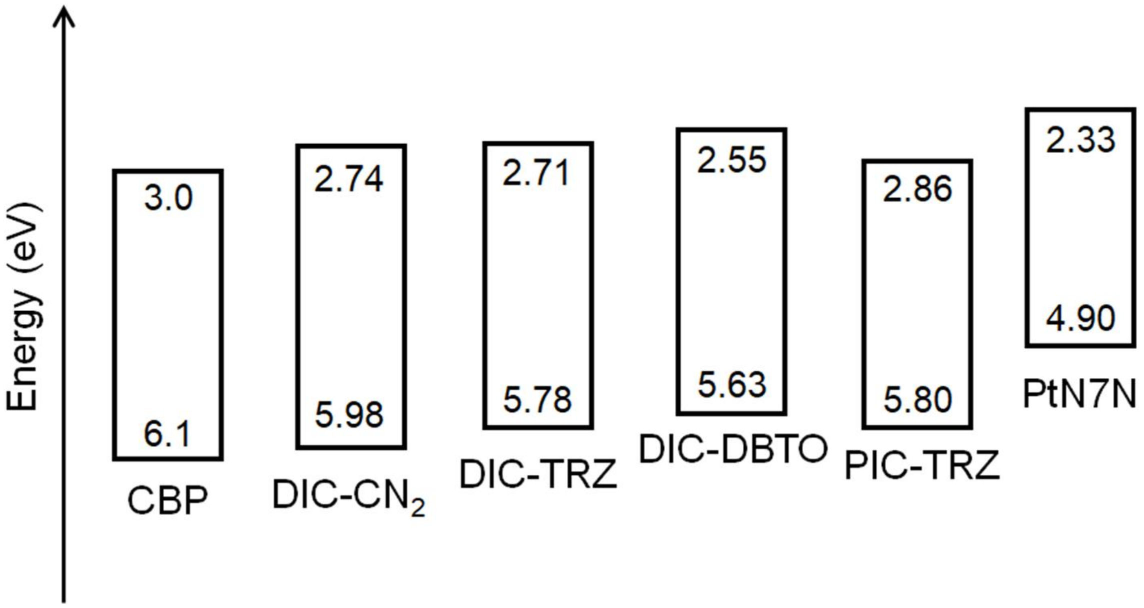

Figure S3. The HOMO and LUMO levels of the host materials (CBP, DIC–TRZ, DIC–CN_2, DIC–DBTO, and PIC–TRZ) and the emitter (PtN7N).

Table S1. Full width at half maximum (FWHM) values of the photoluminescence spectra of the 0–0 band of PtN7N in different host materials such as CBP, DIC–TRZ, DIC–CN_2, DIC–DBTO, TmPyPB, TmPPyTz, Cz–Ph–PMD, Cz–Ph–TRZ, Cz–Ph–TRZPy, and PIC–TRZ.

**Host materials**	**FWHM (nm)**
CBP	18.7
DIC–TRZ	28.4
DIC–CN_2	23.2
DIC–DBTO	22.4
TmPyPB	18.7
TmPPyTz	56.8
Cz–Ph–PMD	20.9
Cz–Ph–TRZ	54.5
Cz–Ph–TRZPy	113.9
PIC–TRZ	25.4
